# Progression of phosphine resistance in susceptible *Tribolium castaneum* (Herbst) populations under different immigration regimes and selection pressures

**DOI:** 10.1111/eva.12493

**Published:** 2017-06-14

**Authors:** Michelle A. Rafter, Graham A. McCulloch, Gregory J. Daglish, Gimme H. Walter

**Affiliations:** ^1^ School of Biological Sciences The University of Queensland Brisbane QLD Australia; ^2^ Department of Agriculture and Fisheries EcoSciences Precinct Brisbane QLD Australia

**Keywords:** fitness, insecticide resistance, migration, pleiotropy, polyandry, red flour beetle, Tenebrionidae

## Abstract

Insecticide resistance is an escalating global issue for a wide variety of agriculturally important pests. The genetic basis and biochemical mechanisms of resistance are well characterized in some systems, but little is known about the ecological aspects of insecticide resistance. We therefore designed a laboratory experiment to quantify the progression of phosphine resistance in *Tribolium castaneum* populations subject to different immigration regimes and selection pressures. Mated resistant females were added to originally susceptible populations under two distinct migration rates, and in addition, half of the populations in each migration treatment were exposed to selection pressures from phosphine fumigation. The progression of phosphine resistance was assessed by screening beetles for the resistance allele at *rph*2. Phosphine resistance increased slowly in the low migration treatment and in the absence of selection, as expected. But at the higher migration rate, the increase in frequency of the resistance allele was lower than predicted. These outcomes result from the high levels of polyandry known in *T. castaneum* females in the laboratory, because most of the Generation 1 offspring (86%) were heterozygous for the *rph2* allele, probably because resistant immigrant females mated again on arrival. Phosphine resistance was not fixed by fumigation as predicted, perhaps because susceptible gametes and eggs survived fumigation within resistant females. In terms of phosphine resistance progression in populations exposed to selection, the effect of fumigation negated the difference in migration rates. These results demonstrate how species‐specific traits relating to the mating system may shape the progression of insecticide resistance within populations, and they have broad implications for the management of phosphine resistance in *T. castaneum* in the field. ​We specify and discuss how these mating system attributes need to be accounted for when developing guidelines for resistance management.

## 1 INTRODUCTION

The genetic basis, mode of inheritance and biochemical mechanisms of insecticide resistance are now fairly well characterized in some systems (ffrench‐Constant, 2013; Roush & McKenzie, 1987). But the ecological variables that influence insecticide resistance, particularly those that influence its establishment and increase in populations and thus its geographical spread, remain unclear. Specific unknowns and difficulties include characterizing the rate at which resistance increases under selection, estimating the amount of gene flow required to spread the genes responsible for resistance and to establish them in a new locality and determining how selection and migration rates interact with one another in terms of the spread of resistance and the establishment of resistance in new localities. Factors that influence the rate of resistance evolution (or rate of change of allele frequencies) in the field include i) initial resistance allele frequencies, which are usually unknown in cases of field resistance; ii) dominance of the alleles responsible for resistance; iii) the relative fitness of genotypes and iv) population structure and dynamics (i.e., the subdivision of populations into smaller breeding units and the amount of gene flow among them) (Roush & McKenzie, 1987).

How resistance alleles persist in populations in the absence of selection is also unclear. Pleiotropic effects of the resistance alleles may impose fitness costs on the organisms in the absence of selection, and such disadvantages may work against the local establishment of resistance alleles and thus the spread of those alleles to fixation (Berticat et al., 2008; Hall, Gubbins, & Gilligan, 2004). But substantial disadvantages of this nature to resistant individuals seem to be the exception rather than the rule in arthropods (Castaneda et al., 2011; Daglish, Nayak, Pavic, & Smith, 2015; Lopes, Sucena, Santos, & Magalhaes, 2008; Roush & McKenzie, 1987). Further, resistance‐associated mutations are often present before the insecticide is ever applied widely in the field (Gould et al., 1997; Hartley et al., 2006; Wenes et al., 2006), so they may have had some other function (perhaps locally or sporadically) prior to their role in resistance (ffrench‐Constant, 2013). As a consequence, resistance‐associated mutations may not always carry a cost in the absence of the insecticides that these organisms encounter later (Arnaud & Haubruge, 2002; ffrench‐Constant, 2007, 2013; Santos‐Amaya et al., 2017). To assess potential fitness disadvantages that result from the resistance alleles carried by organisms, researchers either have to i) measure components of fitness (i.e., fecundity, development time, fertility, mating ability, movement and resource location capabilities) for each genotype (e.g., Malekpour, Rafter, Daglish, & Walter, 2016) or ii) follow changes in genotypic frequencies in replicate populations held for a number of discrete generations in “population cages,” under which no selection regime is applied (Roush & McKenzie, 1987; Tang et al., 2001; Zhao, Collins, & Shelton, 2010).

Population cage studies are informative but do not allow direct inferences as to how insecticide resistance establishes and spreads in susceptible populations under the influence of migration and selection. Accurate models of the spatiotemporal dynamics of such resistance would be useful to management but require accurate data if they are not going to be based on broad assumptions about fitness, dominance, migration rates and the proportion of populations that escape treatment (Roush & McKenzie, 1987; Tang et al., 2001; Zhao et al., 2010). The aim is for general models that are broadly applicable, but the limited data available (on any system) mean that we cannot yet assess how robust such models are likely to be.

In *Tribolium castaneum* (Herbst) (the red flour beetle), resistance to the grain fumigant phosphine (PH_3_) is coded by two major autosomal genes, which are incompletely recessive (Jagadeesan, Collins, Daglish, Ebert, & Schlipalius, 2012). These two genes (labelled *rph1* and *rph2*) are unlinked and each confers weak resistance independently, but in combination act synergistically to confer very strong resistance (431× in *T. castaneum*) (Jagadeesan et al., 2012). Weak PH_3_ resistance conferred by the *rph1* allele is said to have no pleiotropic effects when assessed using the population cage method (Daglish et al., 2015; Jagadeesan, Fotheringham, Ebert, & Schlipalius, 2013; Jagadeesan et al., 2012). PH_3_ resistance conferred by the *rph2* allele is, however, reported to incur a significant fitness cost relative to the wild‐type allele in cage populations (Jagadeesan et al., 2013). *Tribolium castaneum* has some unusual life history traits for a small insect, including that it is long‐lived as an adult, up to a year (Nilsson, Fricke, & Arnqvist, 2002). Generations and life stages thus overlap within the habitat provided by stored grains (Fedina & Lewis, 2008). The females are highly polyandrous in the laboratory (Fedina & Lewis, 2008), but mating rates and the share of paternity across male sires of multiply mated females in the field remain unknown (Demont et al., 2014). Food resources of this species are discrete and patchily distributed across the landscape, and population densities and migration rates between resource patches are also unknown, but adults of both sexes actively disperse across the agricultural landscape through flight (Ridley et al., 2011).

We therefore designed a laboratory experiment to investigate the effect of beetles, homozygous for strong resistance, migrating at different rates into initially susceptible *T. castaneum* populations, and to examine the influence of selection on the frequency and persistence of resistance in those populations. We thus addressed two questions. First, in the absence of selection, does the *rph2* resistance allele increase in populations at the rate predicted, or do genetic (e.g., pleiotropic) or ecological factors (e.g., polyandry) influence the progression of PH_3_ resistance? Second, how rapidly does strong selection (PH_3_ fumigation) drive the fixation of the *rph2* resistance allele, and does the frequency of *rph2* resistance prior to selection affect the rate at which fixation occurs?

## 2 MATERIALS AND METHODS

### 2.1 *Tribolium castaneum* culturing and genotyping

A strongly PH_3_‐resistant strain (QTC931) that carries the G135S resistance allele (Schlipalius et al., 2012) and a PH_3_‐susceptible strain (QTC279) were used in the experiment. The PH_3_‐resistant strain originated as a resistant field sample collected in 2000 from southern Queensland, Australia, and was then exposed to selection in the laboratory to ensure homozygosity for resistance (Jagadeesan et al., 2012). Adults of this strain are about 431× times more resistant than susceptible adults (Jagadeesan et al., 2012). The PH_3_‐susceptible strain was originally developed as a pyrethroid‐resistant strain from a field sample collected in 1984 from southern Queensland (Collins, 1990), but is fully susceptible to PH_3_ (G.J. Daglish, unpublished data). Cultures of these strains were reared on wholemeal flour + yeast (20:1 w/w) at 30°C and 60% relative humidity (r.h.), and these were the conditions used throughout the experiment.

### 2.2 Migration of resistant beetles into susceptible populations with no PH_3_ selection

The initial parental generation in each replicate population comprised 200 unsexed susceptible beetles (2 weeks posteclosion) in a 0.5‐L jar with 400 g of rearing medium (i.e., 0.5 adults/g). Twenty jars thus prepared were divided into two groups of 10 replicates. In the low migration rate treatment, two resistant females were added to each of 10 jars, whereas the high migration rate treatment had 20 resistant females added to each of the remaining 10 jars. Laboratory studies indicate that *T. castaneum* beetles reach sexual maturity 3 days after eclosion (Arnold, Cassey, & White, 2016; Sokoloff, 1974). Data on the reproductive status and sex ratio of dispersing beetles sampled as they migrate from infested grain in the field indicate that the sexual ratio of *T. castaneum* does not deviate significantly from 1:1 and that virtually all females had mated and were able to produce numerous offspring (Ridley et al., 2011). Therefore, mated female immigrants that were at least 2 weeks posteclosion were transferred into experimental populations. Using mated females also ensured that resistance genes were introduced into each test population. Beetles in each of the 10 replicates were allowed to mate and oviposit for 2 weeks, and then, all adult beetles were removed from the jars by sieving the flour, leaving only larvae and eggs in each replicate container. Parental adults were removed in this way because the adults are long‐lived and overlap of multiple generations would have ensued otherwise (Sokoloff, 1974).

**Figure 1:**
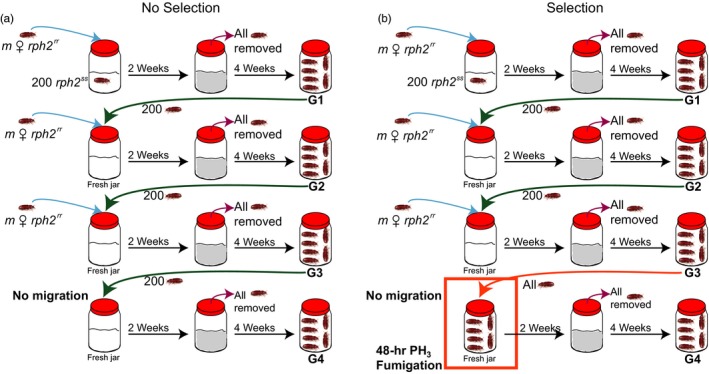
Schematic diagram of the methods for (a) no selection and (b) selection treatments at both the low and high migration rates over the first four generations of the seven‐generation experiment. Blue arrows indicate when two (low) or 20 (high) *rph2* ^*rr*^ females were added to each generation; purple arrows indicate the point at which all adult beetles were removed from jars after 2 weeks of mating and oviposition, leaving only eggs and larvae (as indicated by the grey flour) in the jars. Green arrows indicate when 200 beetles were transferred into a fresh culture jar. Orange arrows in the selection treatments (b) indicate when all beetles from generation 3 were transferred into a fresh culture jar for exposure to a 48‐hr PH_3_ fumigation (orange box). These steps repeat from generation 4 to generation 7 with an additional 48‐hr PH_3_ fumigation at generation 6 in the selection treatments

Once the first generation of offspring emerged as adults (G1), 4 weeks after removal of the original parental adults, 200 adult G1 beetles from each replicate were then transferred into a new jar containing fresh culture medium (Figure 1), and the remaining G1 adults were transferred into ethanol for genetic screening. The same was performed for each successive generation. At this point, the required number of resistant female immigrants was added to each replicate at the specified migration rate. This process (Figure 1) was repeated for seven generations. No migration was permitted at the third and sixth generations in any treatments, for this was when the populations undergoing selection were exposed to PH_3_ (see Section 2.3 below). The original jars were left for 4 weeks so that all of the progeny had emerged as adults, at which point the number of progeny produced in each replicate was assessed. To do this, each jar was placed into a bucket with a lid and a paper ramp was placed on the medium. The beetles readily walked up the paper ramp and fell into the bucket enclosing the jar, but could not return. The beetles in the bucket were weighed together and the total number was calculated by dividing this weight by the known weight of 100 beetles (which was calibrated for each generation of beetles). Beetles were stored in 100% ethanol for subsequent molecular screening for resistance (Section 2.5 below).

### 2.3 Migration of resistant beetles into susceptible populations with PH_3_ selection

This treatment was a repeat of that above was run simultaneously and differed from that above only in that the third and sixth generations were subjected to selection in the form of PH_3_ fumigation (Figure 1). After being weighed to estimate population size in each replicate (at each of these generations), all of the adults (from each particular replicate) were placed in a culture jar containing 400 g of rearing medium 2 days preceding fumigation. Immigrant beetles were not added to these two fumigated generations. The jars to be fumigated were placed in a large‐sealed fumigation chamber (3.24 m^3^) at Hermitage Research Station (Warwick, Queensland, Australia). Mean ambient temperatures during fumigations were 6.5–8.3°C overnight and 25.2–27.6°C during the day over the two fumigation periods (May and October 2014). One‐fifth (0.44 g) of a Pestex^®^ aluminium phosphide tablet (Adama Australia Pty Ltd, St Leonards, Australia) was placed in the fumigation chamber to achieve a sublethal PH_3_ dose (0.135 g/m^3^). This dose should kill 100% of the susceptible and heterozygote genotypes and be sublethal only for the strongly resistant genotypes (Ridley, Magabe, Schlipalius, Rafter, & Collins, 2012). Exposure to PH_3_ was terminated after 48 hr, when the jars were held in a CT room at 30°C and 60% r.h. for 2 weeks before mortality was assessed (to avoid errors from the temporary knockdown effect that PH_3_ also generates). The number of beetles surviving fumigation was assessed by weighing them together (or by counting them if the number of survivors was low), with these beetles then being stored in 100% ethanol for subsequent molecular screening for resistance. Dead adults were also removed from the fumigated jars and stored in 100% ethanol. Dead beetles were not weighed but the number of dead was estimated by subtracting the weight of beetles surviving fumigation from the initial population weight of each replicate. The efficacy of fumigation in the selection treatment was judged by molecular screening of both surviving and dead beetles. This process served as an internal control to assess whether fumigations were killing beetles of the expected genotypes. After removal of the adults, the jars of rearing medium were retained and the surviving eggs provided the basis of the next generation.

### 2.4 Design of restriction assay for the *rph2* resistance allele

To assess the progression of PH_3_ resistance in laboratory populations, we designed a restriction assay to determine whether individual beetles carried the *rph2* resistance allele. Although at least two genes have been implicated in PH_3_ resistance, only one of these, *rph2*, has been fully characterized (Schlipalius et al., 2012). We amplified a 580‐bp region of this gene from 10 individuals from both the strongly resistant strain and the fully susceptible strain using the primers DLD530F (TGCAATCGGCCATTCGAAAC; Malekpour et al., 2016) and DLDi589R (ATTGTCCACGCTTATGCCAC). PCRs were carried out using 12 μl reaction mixture containing 1× MyTaq (Bioline) buffer, 0.2 μM each of forward and reverse primer, 0.3 units of Taq polymerase and 2 μl of template DNA. PCR cycling conditions consisted of an initial denaturation step of 95°C for 2 min, followed by 35 cycles of 95°C for 25 s, 57°C for 30 s and 72°C for 1 min, with a final extension step of 72°C for 10 min. PCR products were cleaned with ExoSAP (Glenn & Schable, 2005), with sequencing reactions performed using a capillary ABI3730 Genetic Analyzer (Applied Biosystems).

Geneious 7.1.6 (Kearse et al., 2012) was used to identify restriction sites in the sequences. The restriction enzyme Taqα 1 (Thermo Fisher Scientific) was selected as it cut the susceptible sequence only once (producing bands of 230 bp and 350 bp in length), whereas the strongly resistant sequence was cut twice (producing bands of 140 bp, 210 bp and 230 bp).

### 2.5 DNA extraction, amplification and restriction digest

Genomic DNA was extracted from 96 individuals per generation with 10% Chelex solution (Walsh, Metzger, & Higuchi, 1991). Prior to PCR amplification, the extracted DNA was diluted 1/25 with H_2_O to remove any contaminants. A 600‐bp fragment of the *rph2* gene was amplified following the protocol outlined above. PCR products were then digested with the restriction enzyme Taqα 1 in a total volume of 20 μl, with about 0.5 μg of DNA (10 μl of PCR product) and 1U of the restriction enzyme in the recommended buffer. Restriction fragments were visualized either by gel electrophoresis (120V for 40 min in 2% agarose with ethidium bromide staining) or by capillary microchip electrophoresis (MultiNA, Shimadzu), with each individual beetle recorded as being homozygous susceptible (*rph2* ^*ss*^), homozygous resistant (*rph2* ^*rr*^) or heterozygous (*rph2* ^*rs*^).

We screened five replicate populations of those not exposed to PH_3_ selection for each of the seven generations that the experiment lasted (96 individuals per replicate for each generation). Likewise for populations that were exposed to PH_3_ selection, we screened 96 individuals per replicate from G1, G2, G4, G5 and G7, but also screened 96 individuals of both the surviving and dead beetles per replicate in G3 and G6 (to assess the extent of the selection pressure imposed by the PH_3_ fumigation event). Because fewer than 96 beetles survived the fumigations under the low immigration rate (see 3), we screened all the surviving beetles from the 10 replicates in this treatment.

For each generation, the genotypic frequency of the *rph2* resistance allele was calculated. In the populations exposed to PH_3_ fumigation, the genotypic frequency of the *rph2* allele in G3 and G6 was estimated by screening the frequency of *rph2* in the surviving and dead beetles and scaling the frequency according to the relative abundance of each.

### 2.6 Predicting progression of the *rph2* resistance allele

We estimated progression of the *rph2* resistance allele due to migration using the formula: p_x(t+1)_ = p_xt_ [1‐*m*] + p_y_ *m* (p_x_ represents the proportion of the *rph2* resistance allele in the recipient populations, p_y_ represents the proportion of the *rph2* resistance allele in the donor population, and *m* represents the migration rate between populations). The extent of re‐mating that would occur after resistant females were migrated into the susceptible populations was unknown; therefore, our initial prediction assumed that mated female immigrants did not re‐mate and that 50% of the beetles in the recipient population would be mated females. Therefore, *m* was calculated to be 0.02 for the low migration rate (two mated resistant females into a population of 100 mated susceptible females) and 0.17 for the high migration rate (20 mated resistant females into a population of 100 mated susceptible females). For the populations exposed to fumigation, we assumed that only strongly resistant eggs and sperm within resistant females would survive fumigation of those females (as it is unknown whether the fumigant can kill susceptible genotype gametes within the female reproductive tract); therefore, it was predicted that the *rph2* allele would be fixed in all populations in the selection treatment at G4.

The results of our laboratory experiments caused us to re‐evaluate our initial assumptions (see 4). We therefore developed a *post hoc* prediction to assess whether incorporating species‐specific traits relating to the mating system would lead to a more accurate prediction of the progression of the *rph2* resistance allele in the populations that underwent selection. Specifically, we modified our prediction to incorporate several new assumptions: i) resistant females re‐mate after migration and ii) all gametes and eggs inside resistant females survive fumigation. Our results suggest that most (86%) of the offspring produced by immigrant females were from matings that occurred after immigration, so we halved the migration rates to *m = *0.01 and 0.09, to reflect more accurately the migration rate of the *rph2* resistance allele. To predict the frequency of the *rph2* resistance allele in G4 and G7 under the scenario that all gametes and eggs inside resistant females survive fumigation, we used the formula $p_{x(t+1)}=p_{x}^{2}+1.5\times \left(p_{x}q_{x}\right)+0.5\times q_{x}^{2}$ (q_x_ represents the proportion of the nonresistant *rph2* allele in the population).

### 2.7 Statistical analysis

Repeated‐measures ANOVAs were run for population size and *rph2* resistance allele proportion data over the course of the experiment with a Huynh–Feldt or Greenhouse–Geisser correction applied if the assumption of sphericity was violated (IBM 2015). The frequencies of *rph2* resistance alleles at the generations following selection were compared across the two migration rate treatments by means of *t* tests on the raw data. A chi‐square test was used to determine whether genotype frequencies observed at each generation were consistent with Hardy–Weinberg equilibrium.

## 3 RESULTS

### 3.1 Populations with immigration and no PH_3_ selection

**Figure 2:**
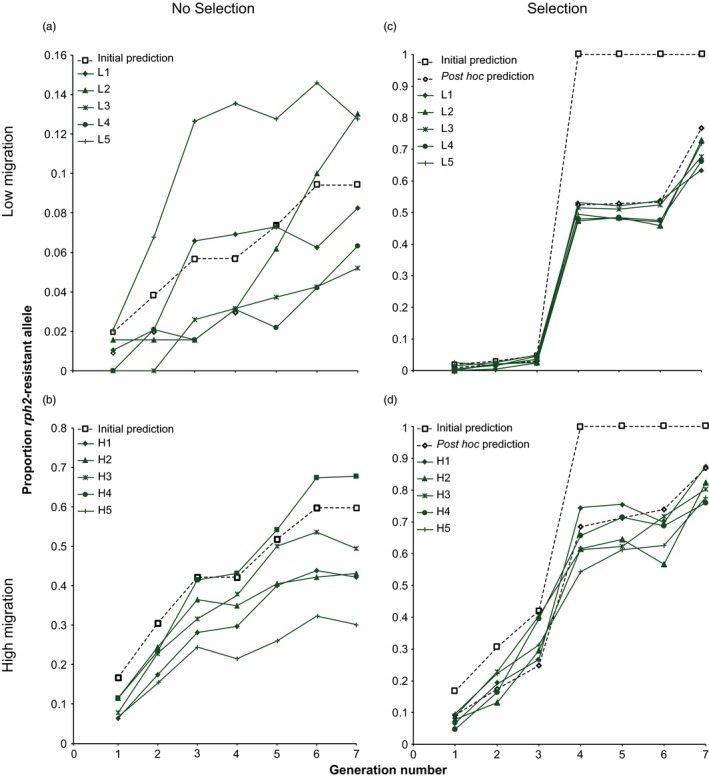
The recorded (green lines) and predicted (dashed lines) frequencies for the *rph2* resistance allele across seven generations for (a) no selection treatment, low migration rate; (b) no selection treatment, high migration rate; (c) selection treatment, low migration rate; and (d) selection treatment, high migration rate. Black hollow squares represent initial predictions in which immigrant resistant females do not re‐mate within the population in which they establish and that only strongly resistant eggs and gametes survive fumigation within resistant females, with black hollow diamonds representing *post hoc* predictions in which the immigrant resistant females re‐mate after immigration, and that all gametes and eggs inside resistant females survive fumigation

**Figure 3:**
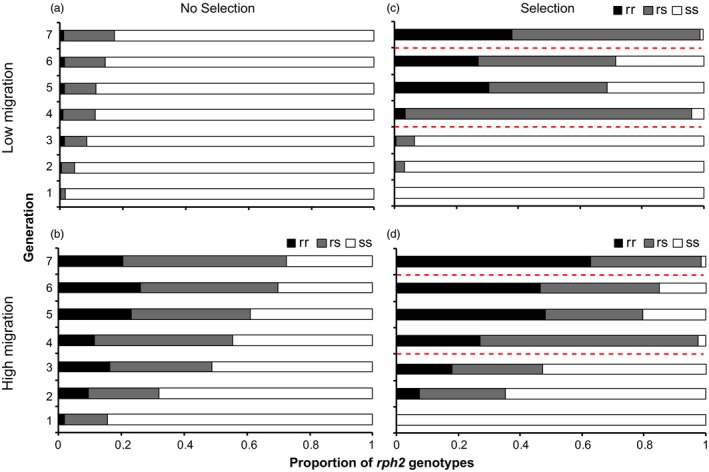
Proportions of *rph2* genotypes in each of seven generations of *Tribolium castaneum* beetles for (a) no selection treatment, low migration rate; (b) no selection treatment, high migration rate; (c) selection treatment, low migration rate; and (d) selection treatment, high migration rate (*n* = 5 replicates in each treatment). Black bars represent the *rph2* ^rr^ genotype, grey bars the *rph2* ^rs^ genotype and white bars the *rph2* ^ss^ genotype. The dashed red lines represent PH_3_ fumigation

At the low migration rate without PH_3_ selection, the resistance allele of *rph2* was detected in three of the five populations in G1 (coded L1, L2 and L5), and by G3, it was detected in all populations (Figure 2a). The frequency of the resistance allele of *rph2* increased by an average of 1.3% per generation (range 0.7%–1.9%), and by G7, the average frequency was 9.1% (range 5.2%–13.0%) (Figure 2a), which was similar to prediction (9.4%). After seven generations, most beetles remained homozygous for the susceptible allele of *rph2* (i.e., *rph2* ^ss^ = 75.5%–89.6%) with the proportion of *rph2* ^rr^ beetles very low (between 0.0% and 2.1%; Figure 3a; Table S2).

At the high migration rate, the resistance allele of *rph2* was detected in all populations screened at G1 (Figure 2b). Most of the beetles in G1 with the resistance allele of *rph2* were heterozygous (*rph2* ^*rs*^) for this allele (86%; Table S2), indicating that the resistant female immigrants re‐mated after entering the population. The frequency of the resistance allele of *rph2* increased by an average of 6.6% per generation (range 4.3%–9.7%), with the frequency at G7 of 46.5% (range 30.2%–67.7%) (Figure 2b), slightly lower than predicted (60%). The frequency of the resistance allele of *rph2* was statistically higher than the frequencies recorded in the low migration rate over seven generations (F_1.51_ = 20.59, *p *<* *.001, Greenhouse–Geisser correction). After seven generations, 20.5% of the beetles were *rph2* ^*rr*^ (range 8.3%–37.5%; Figure 3b).

Estimated population sizes were not constant across generations through the course of the experiment in the treatments with no selection imposed (*F* = 58.66, *df* = 5.11, *p *<* *.001, Huynh–Feldt correction), but the pattern of population size fluctuation was consistent across the two immigration rate treatments, and the population size differences were not statistically significant (*F* = 2.46, *df* = 5.11, *p *=* *.38, Huynh–Feldt correction) (see Table S1). Allele frequencies were in Hardy–Weinberg equilibrium for the two generations in which there was no immigration (i.e., G4 and G7), but allele frequencies deviated significantly from Hardy–Weinberg equilibrium in the generations with immigration (Table S4).

### 3.2 Populations with immigration and PH_3_ selection

**Figure 4:**
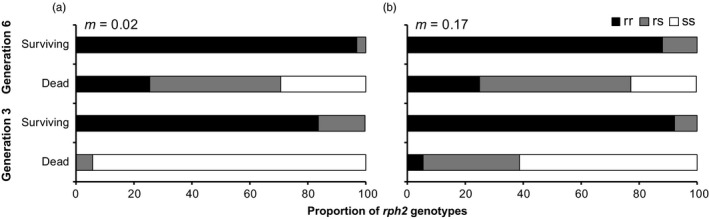
Proportions of *rph2* genotypes of beetles that were alive or dead after PH_3_ fumigation at generations 3 and 6 from (a) the low migration rate treatment and (b) the high migration rate treatment. Black bars represent the *rph2* ^rr^ genotype, grey bars the *rph2* ^rs^ genotype and white bars the *rph2* ^ss^ genotype

The average percentage mortality after fumigation at G3 was between 98.9% and 99.8% for the low migration rate and between 82.0% and 96.2% for the high migration rate, whereas at G6 the percentage mortality was between 96.2% and 98.9% for the low migration rate and between 50.1% and 74.6% for the high migration rate treatment (Table 1). Mortality was statistically lower after the second fumigation (i.e., in G6 relative to G3) for only the higher migration rate (*t*_17.96_ = −0.89, *p *=* *.38; *m *=* *0.17, *t*_16.80_ = 3.09, *p *=* *.007). Most of the beetles that survived fumigation in G3 and G6 were *rph2* ^rr^ (range 83.6%–96.2%), but a small percentage of *rph2* ^rs^ beetles did survive fumigation (3.8%–16.4%; Figure 4; Table S3). No *rph2* ^ss^ beetles survived either fumigation (Figure 4).

**Table 1:** End‐point mortality (%) (mean, range, ± 1 SE) and the mean number of *Tribolium castaneum* adults that survived (±1 SE) (in each of two generations of the populations concerned) following a 48‐hr selection event (phosphine fumigation) at a target rate of 0.135 mg/L of phosphine gas. The numbers of beetles that were fumigated are also given

Migration rate	Generation 3			Generation 6		
	Mean number of beetles fumigated	Mortality (%)	Beetles surviving	Mean number of beetles fumigated	Mortality (%)	Beetles surviving
Low	4,687.3 ± 253.8	99.3 (98.9–99.8) ± 0.1	32.4 ± 5.9	5,079.1 ± 246.8	97.7 (96.2–98.9) ± 0.3	115.8 ± 19.4
High	6,457.3 ± 304.5	85.9 (82.0–96.2) ± 1.3	955.9 ± 109.2	6,595.9 ± 231.6	66.2 (50.1–74.6) ± 2.5	2,194.0 ± 113.1

Although the proportion of *rph2* resistance alleles in G4 increased substantially in both the high and low migration treatments, it did not go to fixation as predicted (Figure 2c,d), with an average frequency of 49.9% (range 47.4%–53.1%) for the low migration treatment and 62.4% (range 54.7%–69.3%) for the high migration treatment. The average frequency of the *rph2* resistance allele at G4 was statistically higher in the high migration treatment (*t*_6.18_ = −6.41, *p *=* *.0006). Most of the beetles from G4 were *rph2* ^*rs*^ (92.7% for the low migration treatment, 70.6% for the high migration treatment), with smaller proportions of *rph2* ^rr^ (3.5% for the low migration, 27.1% for the high migration) and *rph2* ^ss^ (3.8% for the low migration treatment*,* 2.3% for the high migration treatment; Figure 3c,d). The frequency of the resistance allele of *rph2* again increased substantially for both low and high migration treatments in G7 (after the G6 fumigation), but it still did not reach fixation by G7 (Figure 2c,d). The average frequency of the *rph2* resistance allele at G7 was again statistically higher in the high migration treatment (*t*_4.53_ = −5.85, *p *=* *.003). After five generations of migration, and two fumigations, less than half of the beetles (38%) in the low migration treatment were *rph2* ^rr^ with most of the beetles (61%) *rph2* ^rs^ (Figure 4c,d). A larger proportion of beetles in the high migration treatment were *rph2* ^rr^ (63%), but again substantial numbers were *rph2* ^rs^ (36%; Figure 3c,d). Modified *post hoc* predictions matched up much more closely with our experimental results (Figure 2c,d). For example, at G4 the frequency of the resistance allele of *rph2* was predicted to be 52.4% for the low migration treatment (average experimental frequency 49.9%) and 68.4% for the high migration treatment (average experimental frequency 62.4%).

Average population sizes in the treatments exposed to fumigation were similar to those observed in the control treatments (with no selection from PH_3_ fumigation), but with a notable decrease in population sizes in G4 and G7 after fumigations (Table S1). Population sizes were higher in the high migration rate treatments (because 18 more beetles were introduced at each generation that was not fumigated), but not significantly so over the duration of the experiment (*F* = 1.89, *df* = 2.17, *p *=* *.169, Greenhouse–Geisser correction), undoubtedly because each subsequent generation was started with 200 beetles from the previous generation.

## 4 DISCUSSION

This study demonstrates the power of laboratory experiments in assessing how allelic interactions, migration and selection interact to shape the progression of resistance in previously susceptible insect populations. In *T. castaneum*, resistance to PH_3_ is coded by two major autosomal genes that are incompletely recessive (Jagadeesan et al., 2012). Each of these two resistance loci (*rph1* and *rph2*) confers weak resistance when homozygous for a resistance allele without the other locus being homozygous for resistance. In combination, though, when both are homozygous for resistance, these loci act synergistically to confer very strong resistance (Jagadeesan et al., 2012). In the following sections, we examine how traits such as mating system attributes and fitness costs influence the ecology of the organisms and thus impact upon the progression of one of the PH_3_ resistance alleles, *rph2* (G135S)*,* in the presence and absence of strong selection pressures (PH_3_ fumigation).

### 4.1 Progression of PH_3_ resistance in the absence of selection

Resistance alleles within the experimental populations were detectable, and increased steadily at both migration rates, even in the absence of PH_3_ selection (Figure 2a,b). Although a significant amount of stochastic variation among populations did affect allelic frequencies, clear patterns were still evident. The progression of the resistance allele of *rph2* in the low migration treatment generally followed prediction, but its frequency was notably lower than expected at the high migration rate, probably as a result of the high levels of polyandry known in *T. castaneum* females in the laboratory (Fedina & Lewis, 2008; Lewis, 2004; Lewis, Kobel, Fedina, & Beeman, 2005), a feature confirmed recently in field populations (MAR, unpublished data). *Tribolium castaneum* females store sperm in a group of ducts contained within the membranous spermatheca located off the bursa copulatrix (Sinha, 1953; Surtees, 1961). With multiple matings, sperm is initially stratified in storage, with the last male to copulate experiencing a relatively higher paternity share (Droge‐Young, Belote, Perez, & Pitnick, 2016; Lewis & Jutkiewicz, 1998). This pattern of sperm stratification is reflected in our results, as 86% of the offspring produced by the resistant immigrant females in G1 were a result of matings that occurred after immigration. This implies that only a small proportion of the offspring produced by these strongly resistant females had strong resistance to PH_3_, leading to a slower progression of PH_3_ resistance in the populations than would have occurred if the resistant females had not re‐mated on arrival.

The migration rate of beetles in the field is difficult to quantify, although recent studies suggest *T. castaneum* (Ridley et al., 2011; Semeao et al., 2012) and other grain beetles (Ridley et al., 2016) actively disperse by flight. Beetles carrying the *rph2* resistance allele, however, have been shown to have a lower flight propensity compared to susceptible beetles (Malekpour et al., 2016). The progression of PH_3_ resistance would be notably slower if immigrant females were not strongly resistant to PH_3_, and likewise if females had mated with only susceptible males prior to immigration (however, given the extent of re‐mating that occurs after immigration, this is unlikely to greatly affect the progression of PH_3_ resistance). The proportion of homozygous resistant beetles (*rph2* ^*rr*^) in the low migration treatment remained low (range 0%–2.1%) over the duration of the experiment (Figure 3a), but was notably higher in the high migration treatment (range 8.3%–37.5%) (Figure 3b). Strong PH_3_ resistance should therefore develop only slowly in a field population exposed to immigrant insects but not subject to PH_3_ treatment, but would develop more rapidly if weak resistance was already fixed in a population (Schlipalius et al., 2008).

### 4.2 Progression of PH_3_ resistance in the presence of strong selection

The frequency of the resistance allele at *rph2* increased dramatically in all populations after PH_3_ selection was applied (Figure 2c,d). In G4, the average proportion of the resistance allele at *rph2* was 0.50 and 0.62 in the low and high migration treatments, respectively, compared with 0.06 and 0.33 in the corresponding no selection treatments (Figure 2). The effect of selection almost seemed to negate the difference in migration rates but, contrary to initial prediction, the resistance allele at *rph2* was not entirely fixed by fumigation. A small proportion of *rph2* ^rs^ beetles survived the initial selection event in both the low (23.5%) and the high migration (7.7%) treatments, but the presence of these survivors alone did not explain the lower‐than‐predicted proportion of the resistance allele at *rph2* in G4. Likewise, a smaller proportion of *rph2* ^rs^ beetles survived the G6 selection (3.3% for the low migration rate, 11.8% for the high migration rate), but again this failed to explain the lower‐than‐expected proportion of the resistance allele at *rph2* in G7.

The degree of tolerance to PH_3_ varies significantly across the different stages of an insect's life cycle (Nakakita & Winks, 1981; Price & Mills, 1988; Rajendran, 1992), and it seems likely that this differential may have contributed to our results. *Tribolium castaneum* eggs may be substantially more tolerant than adults to PH_3_ (Hole, Bell, Mills, & Goodship, 1976; Price & Mills, 1988; Rajendran, 1992), so eggs that are *rph2* ^rs^ (or possibly even *rph2* ^ss^) may survive a dose of PH_3_ that would kill adult beetles with these haplotypes. Adult beetles were transferred into new jars 2 days prior to fumigation, with eggs laid during this time potentially contributing to the following generation. The increased numbers of eggs laid by G3 females (in each replicate) prior to fumigation are unlikely to have made a significant contribution to the number of individuals in the subsequent G4 replicates. The direct correlation between the population sizes in G4 and the number of beetles surviving fumigation in G3 (Figure S1) demonstrates that the survivors from the G3 fumigation, rather than eggs laid prior to fumigation, are the primary contributor to G4.

The increased tolerance of gametes (and eggs) inside resistant females at the time of fumigation may therefore explain the lower‐than‐predicted increase in the *rph2* resistance allele, with resistant females having the potential to carry both resistant and susceptible gametes. If the tolerance of susceptible gametes is high enough that they survive fumigation inside resistant females (or the female reproductive tract protects these gametes from the full effects of fumigation), they would be expected to be the major contributor to the following generation given the high proportion of the *rph2* ^ss^ beetles in the populations. The proportion of the resistance allele at *rph2* after fumigation should therefore be correlated with the proportion of the same resistance allele at *rph2* in populations prior to fumigation, which appears to be the case in our experiment, with the proportion of the resistance allele at *rph2* higher in the high migration treatment after fumigation than in the low migration treatment. Indeed, when we assume that all gametes survive fumigation inside resistant females (*post hoc* predictions; Figure 2c,d), our experimental results fit much more closely with our predictions for both migration rates. Our results still remain slightly lower than predicted, potentially because a small proportion of *rph2* ^*rs*^ beetles survived fumigation. This demonstrates how the incorporation of traits specific to the mating system of species in question can lead to more accurate predictions of the progression of insecticide resistance genes in populations.

The level of mortality as a result of the G3 fumigation was lower in the high migration treatment than in the low migration treatment, consistent with the molecular results showing a much higher proportion of the resistance allele at *rph2* in the high migration treatment. While mean mortality as a result of the G6 fumigation decreased for both the low and high migration treatments, it still remained high, contrary to our prediction that there would be little or no mortality due to the resistant alleles being fixed after the fumigation at G3. The mortality levels after the G6 fumigation were substantially higher in the low migration treatment, which is consistent with the molecular results showing a higher level of the resistance allele at *rph2* at G5 in the high migration treatment (67.0%) compared to the low migration treatment (49.6%). This also confirms the results of the molecular screening, which indicate that the level of PH_3_ resistance in the population prior to fumigation greatly impacts the rate at which PH_3_ resistance progresses after fumigation.

### 4.3 Pleiotropic effects of PH_3_ resistance alleles

The steady increase in the frequency of the resistance allele of *rph2* in our laboratory populations, at a similar rate to that predicted in both the low and high migration rates, suggests there is no strong selective fitness disadvantage to having the *rph2* resistance allele (at least in beetles confined entirely within laboratory cultures (see Malekpour et al., 2016)). In laboratory populations of several insect species, including *Spodoptera frugiperda, Plutella xylostella* and *Diatraea saccharalis*, the proportion of resistant individuals is stable in the absence of selective pressures (Santos‐Amaya et al., 2017; Tang, Gilboa, Roush, & Shelton, 1997; Zhang et al., 2014). These studies indicate that natural selection against some resistance alleles is weak under optimal conditions in the laboratory. But additional studies under differing environmental conditions or even under field conditions are required to validate these patterns (Zhang et al., 2014). Any weak selective disadvantage that may be present would potentially be masked by the increase in the resistance allele at *rph2* each generation, with this driven by the immigration of resistant females. These results are largely consistent with previous population cage studies that likewise provide little evidence for any reproductive fitness cost of resistance under laboratory conditions (Daglish et al., 2015; Jagadeesan et al., 2012; Pimentel, Faroni, da Silva, Batista, & Guedes, 2010; Pimentel, Faroni, Guedes, Sousa, & Tótola, 2009; Sousa, Faroni, Pimentel, & Guedes, 2009). However, several recent genetics studies have reported a significant selective fitness disadvantage (in the absence of selection from PH_3_) for individuals homozygous for the resistance allele at *rph2* in both *T. castaneum* and *Sitophilus oryzae* (L.) (the rice weevil) (Jagadeesan et al., 2013; Malekpour et al., 2016; Nguyen, Collins, Duong, Schlipalius, & Ebert, 2016), along with a strong selective fitness advantage of the homozygous resistance genotype at the *rph1* locus in *T. castaneum* (Jagadeesan et al., 2013). The contrasting results between these studies and our results likely reflect a difference in study design. In our study, mated females were introduced at regular intervals, leading to an excess of strongly resistant beetles in each subsequent generation (*rph1* ^rr^/*rph*2^rr^). Any significant selective disadvantage resulting from the *rph2* ^rr^ phenotype in these beetles could be counteracted by a significant selective advantage of the *rph1* ^rr^ phenotype. Notably, in G4 and G7—the two “random mating” generations—there is a slight (but nonsignificant) deficit of beetles homozygous for the *rph2* resistance allele. This suggests that there could be negative pleiotropic effects associated with homozygosity for this allele, but only when beetles are not also homozygous for the *rph1* resistance allele.

In contrast to the results of experiments on beetles confined within the laboratory, studies on field populations have detected fitness cost associated with PH_3_ resistance (Pimentel, Faroni, Tótola, & Guedes, 2007; Pimentel et al., 2009, 2010; Sousa et al., 2009). The pleiotropic effects of insecticide resistance can be more subtle than simply affecting intrinsic growth rates or development times under optimal laboratory conditions (Foster et al., 2003). Therefore, the contrast in between field and laboratory studies may reflect the differences in environmental conditions and selection pressures in these two environments. Less obvious pleiotropic effects on the biology and behaviour of insects (such as their responses to alarm pheromones and flight propensity) also need to be investigated (Foster et al., 2003; Malekpour et al., 2016; McKenzie, 1996) to model and predict insecticide resistance evolution in field populations more accurately.

### 4.4 General conclusions

As expected, the immigration of homozygous resistant individuals and the imposition of fumigant treatments that kill susceptible genotypes (but not resistant ones) increased the frequency of resistant alleles rapidly, at least in otherwise contained populations in the laboratory. However, resistance did not progress as rapidly in either the unselected or selected populations as initially predicted. This was likely due to particular mating system attributes of *T. castaneum*, namely polyandry and the susceptible gametes surviving fumigation inside resistant females. Polyandry, as a mating system attribute, is said to protect populations of *T. castaneum* and other insect species from inbreeding depression (e.g., Michalczyk et al., 2011; Tregenza & Wedell, 2002; Zeh & Zeh, 1996), and this study demonstrates that for *T. castaneum* multiple mating also slows the progression of resistance in populations to the fumigant phosphine. Incorporating these traits into our model greatly improved the accuracy of these predictions, demonstrating the importance of understanding species‐specific mating system attributes when assessing how resistance will develop within populations. Results of this experiment should be incorporated in future PH_3_ resistance management plans and should also inform future models and laboratory experiments of resistance development. The results of this experiment have implications for managing insecticide resistance in general: i) many pest insects disperse actively across agricultural landscapes, and thus, migration without selection is a key component to resistance development and progression within and among discrete populations and ii) polyandry and the survival of susceptible genotypes within resistant females are species‐specific mating system attributes that impact upon resistance progression within populations. Thus, it is a variable that needs to be carefully considered and quantified for each pest insect species with the specifics incorporated into models of resistance development.

## DATA ARCHIVING STATEMENT

Data available from the Dryad Digital Repository: https://doi.org/10.5061/dryad.7t7ph.
