# Supplementary material for: Progression of phosphine resistance in susceptible Tribolium castaneum (Herbst) populations under different immigration regimes and selection pressures
Source: Evol Appl. 2017 Jun 14;10(9):907–18. doi: 10.1111/eva.12493 (PMC5680416; doi:10.1111/eva.12493)
Supplement: Supplementary file 1 [file EVA-10-907-s001.docx]

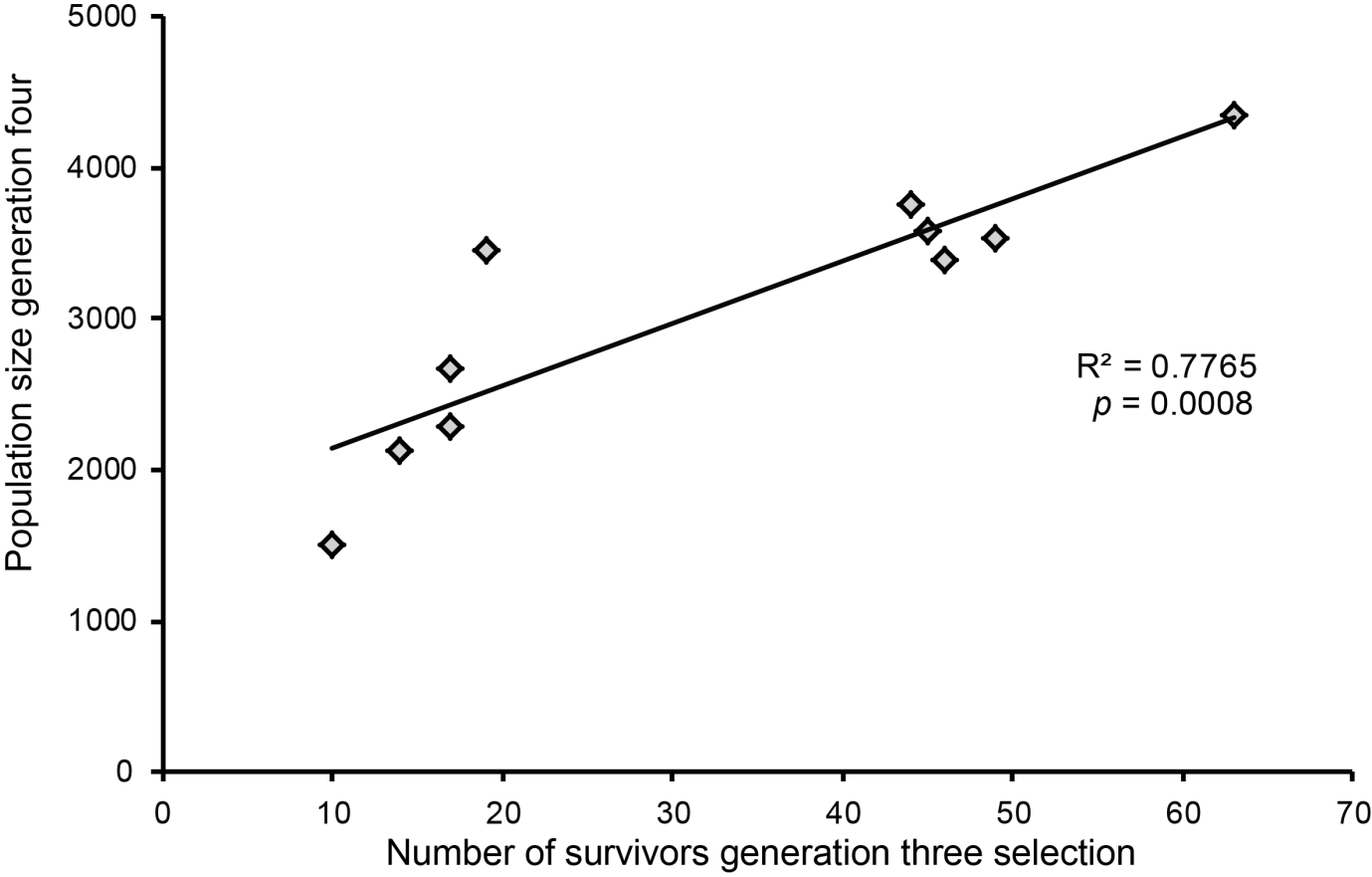


**Figure S1:** Linear regression showing a significant positive relationship between of the number of survivors of the generation three selection event (forty-eight hour PH_3_ fumigation) and the resulting population size of generation four.
