# Supplementary material for: Progression of phosphine resistance in susceptible Tribolium castaneum (Herbst) populations under different immigration regimes and selection pressures
Source: Evol Appl. 2017 Jun 14;10(9):907–18. doi: 10.1111/eva.12493 (PMC5680416; doi:10.1111/eva.12493)
Supplement: Supplementary file 2 [file EVA-10-907-s002.docx]

**Supplementary Table S1.** Estimated mean population numbers (± 1SE) of *Tribolium castaneum* in experimental populations exposed to selection from phosphine fumigation (Selected) or to no such selection (Unselected) across migration and selection treatments (n = 10 for each treatment combination) in each of seven successive generations. Phosphine selection events were imposed at Generations 3 and 6 in the selection treatments and no migration occurred across all treatments in each of these two generations.

| Treatment | Migration | Mean estimated population numbers | | | | | | |
| --- | --- | --- | --- | --- | --- | --- | --- | --- |
|  |  | Generation 1 | Generation 2 | Generation 3 | Generation 4 | Generation 5 | Generation 6 | Generation 7 |
| Unselected | Low | 6643.4 ± 206.4 | 6120.5 ± 123.4 | 6169.1 ± 109.5 | 4622.3 ± 146.8 | 6194.5 ± 162.2 | 7190.5 ± 196.9 | 2391.4 ± 119.4 |
|  | High | 6976.6 ± 139.0 | 5887.7 ± 501.2 | 6609.1 ± 253.7 | 5940.0 ± 153.3 | 7820.0 ± 169.2 | 8035.9 ± 238.8 | 3598.2 ± 271.6 |
| Selected | Low | 6664.3 ± 267.7 | 5845.0 ± 629.9 | 4687.3 ± 452.9 | 3066.4 ± 278.3 | 6554.5 ± 342.1 | 5079.1 ± 246.8 | 3119.1 ± 716.4 |
|  | High | 6940.5 ± 235.6 | 6636.8 ± 487.7 | 6457.3 ± 304.5 | 2349.5 ± 481.7 | 7416.8 ± 288.0 | 6595.9 ± 231.6 | 2764.1 ± 1013.6 |
