# Supplementary material for: Progression of phosphine resistance in susceptible Tribolium castaneum (Herbst) populations under different immigration regimes and selection pressures
Source: Evol Appl. 2017 Jun 14;10(9):907–18. doi: 10.1111/eva.12493 (PMC5680416; doi:10.1111/eva.12493)
Supplement: Supplementary file 3 [file EVA-10-907-s003.docx]

**Table S2.** *Rph2* genotypes recorded over seven generation for the unselected populations.

| **Migration rate** | **Population** | **G1** | | | **G2** | | | **G3** | | | **G4** | | | **G5** | | | **G6** | | | **G7** | | |
| --- | --- | --- | --- | --- | --- | --- | --- | --- | --- | --- | --- | --- | --- | --- | --- | --- | --- | --- | --- | --- | --- | --- |
|  |  | ***rr*** | ***rs*** | ***ss*** | ***rr*** | ***rs*** | ***ss*** | ***rr*** | ***rs*** | ***ss*** | ***rr*** | ***rs*** | ***ss*** | ***rr*** | ***rs*** | ***ss*** | ***rr*** | ***rs*** | ***ss*** | ***rr*** | ***rs*** | ***ss*** |
| ***m* = 0.02** | **L1** | **0** | **2** | **94** | **0** | **4** | **92** | **3** | **6** | **82** | **1** | **11** | **82** | **4** | **6** | **86** | **2** | **8** | **86** | **1** | **14** | **82** |
|  | **L2** | **1** | **1** | **94** | **0** | **3** | **92** | **0** | **3** | **93** | **0** | **6** | **90** | **1** | **10** | **85** | **2** | **15** | **78** | **2** | **21** | **73** |
|  | **L3** | **0** | **0** | **95** | **0** | **0** | **96** | **1** | **3** | **92** | **1** | **4** | **90** | **1** | **5** | **88** | **1** | **6** | **87** | **0** | **10** | **86** |
|  | **L4** | **0** | **0** | **96** | **1** | **2** | **92** | **0** | **3** | **92** | **0** | **6** | **90** | **1** | **2** | **93** | **0** | **8** | **87** | **1** | **10** | **84** |
|  | **L5** | **0** | **4** | **92** | **1** | **11** | **84** | **3** | **18** | **74** | **2** | **22** | **72** | **0** | **24** | **70** | **2** | **24** | **70** | **1** | **22** | **71** |
| ***m* = 0.17** | **H1** | **1** | **11** | **84** | **6** | **17** | **72** | **8** | **31** | **57** | **4** | **32** | **57** | **6** | **38** | **52** | **18** | **26** | **52** | **8** | **42** | **46** |
|  | **H2** | **0** | **15** | **81** | **8** | **28** | **60** | **18** | **24** | **53** | **16** | **40** | **40** | **26** | **44** | **26** | **24** | **55** | **17** | **20** | **55** | **21** |
|  | **H3** | **1** | **10** | **83** | **8** | **16** | **68** | **12** | **30** | **54** | **8** | **41** | **47** | **28** | **20** | **47** | **30** | **26** | **42** | **19** | **43** | **34** |
|  | **H4** | **5** | **12** | **79** | **11** | **22** | **61** | **20** | **38** | **36** | **15** | **52** | **28** | **34** | **36** | **26** | **36** | **56** | **3** | **36** | **58** | **2** |
|  | **H5** | **2** | **18** | **76** | **12** | **23** | **61** | **19** | **32** | **45** | **11** | **45** | **40** | **17** | **43** | **35** | **17** | **47** | **32** | **15** | **51** | **28** |
