# Supplementary material for: Progression of phosphine resistance in susceptible Tribolium castaneum (Herbst) populations under different immigration regimes and selection pressures
Source: Evol Appl. 2017 Jun 14;10(9):907–18. doi: 10.1111/eva.12493 (PMC5680416; doi:10.1111/eva.12493)
Supplement: Supplementary file 4 [file EVA-10-907-s004.docx]

**Table S3.** *Rph2* genotypes recorded over seven generation for the populations that were exposed to selection (phosphine).

| **Migration rate** | **Population** | **G1** | | | **G2** | | | **G3** | | | | | | | **G4** | | | **G5** | | | **G6** | | | | | | | **G7** | | |
| --- | --- | --- | --- | --- | --- | --- | --- | --- | --- | --- | --- | --- | --- | --- | --- | --- | --- | --- | --- | --- | --- | --- | --- | --- | --- | --- | --- | --- | --- | --- |
|  |  |  |  |  |  |  |  | **Survivors** | | |  | **Dead** | | |  |  |  |  |  |  | **Survivors** | | |  | **Dead** | | |  |  |  |
|  |  | *rr* | *rs* | *ss* | *rr* | *rs* | *ss* | *rr* | *rs* | *ss* |  | *rr* | *rs* | *ss* | *rr* | *rs* | *ss* | *rr* | *rs* | *ss* | *rr* | *rs* | *ss* |  | *rr* | *rs* | *ss* | *rr* | *rs* | *ss* |
| *m* = 0.02 | L1 | 0 | 0 | 94 | 1 | 2 | 93 | 16 | 1 | 0 |  | 0 | 3 | 93 | 1 | 88 | 6 | 24 | 45 | 27 | 85 | 2 | 0 |  | 20 | 46 | 30 | 45 | 50 | 1 |
|  | L2 | 0 | 0 | 93 | 0 | 1 | 95 | 37 | 8 | 0 |  | 0 | 3 | 90 | 8 | 86 | 2 | 35 | 30 | 31 | 92 | 4 | 0 |  | 26 | 48 | 22 | 26 | 70 | 0 |
|  | L3 | 0 | 3 | 93 | 0 | 5 | 91 | 32 | 12 | 0 |  | 0 | 8 | 88 | 4 | 91 | 1 | 28 | 42 | 26 | 89 | 4 | 0 |  | 28 | 42 | 25 | 36 | 58 | 2 |
|  | L4 | 0 | 1 | 95 | 0 | 3 | 91 | 11 | 3 | 0 |  | 0 | 6 | 86 | 2 | 88 | 6 | 31 | 30 | 34 | 57 | 1 | 0 |  | 24 | 42 | 30 | 31 | 65 | 0 |
|  | L5 | 0 | 5 | 91 | 0 | 3 | 93 | 35 | 14 | 0 |  | 0 | 7 | 86 | 2 | 91 | 3 | 28 | 36 | 32 | 93 | 3 | 0 |  | 24 | 39 | 33 | 44 | 50 | 2 |
| *m* = 0.17 | H1 | 4 | 10 | 80 | 3 | 27 | 60 | 84 | 12 | 0 |  | 4 | 26 | 64 | 12 | 81 | 3 | 32 | 39 | 25 | 86 | 10 | 0 |  | 16 | 36 | 44 | 65 | 28 | 3 |
|  | H2 | 1 | 4 | 85 | 3 | 18 | 73 | 92 | 4 | 0 |  | 2 | 22 | 74 | 27 | 68 | 1 | 49 | 34 | 13 | 83 | 12 | 0 |  | 30 | 56 | 8 | 73 | 22 | 1 |
|  | H3 | 1 | 15 | 78 | 6 | 32 | 58 | 91 | 5 | 0 |  | 7 | 44 | 40 | 29 | 64 | 3 | 46 | 35 | 15 | 88 | 7 | 0 |  | 24 | 50 | 22 | 58 | 38 | 0 |
|  | H4 | 5 | 22 | 66 | 16 | 30 | 50 | 89 | 7 | 0 |  | 9 | 39 | 48 | 37 | 59 | 0 | 58 | 21 | 17 | 82 | 14 | 0 |  | 32 | 48 | 16 | 51 | 44 | 1 |
|  | H5 | 0 | 24 | 64 | 7 | 24 | 65 | 87 | 9 | 0 |  | 4 | 26 | 66 | 25 | 67 | 4 | 46 | 22 | 28 | 81 | 13 | 0 |  | 18 | 59 | 19 | 54 | 41 | 1 |
