# Supplementary material for: Progression of phosphine resistance in susceptible Tribolium castaneum (Herbst) populations under different immigration regimes and selection pressures
Source: Evol Appl. 2017 Jun 14;10(9):907–18. doi: 10.1111/eva.12493 (PMC5680416; doi:10.1111/eva.12493)
Supplement: Supplementary file 5 [file EVA-10-907-s005.docx]

**Table S4.** Results of a χ^2^ test for deviation from Hardy-Weinburg equilibrium for the *m =* 0.17 selection treatment. Bold values are significant a *P* <0.05.

| Generation | *P* value |
| --- | --- |
| 1 | **<0.001** |
| 2 | **0.002** |
| 3 | **<0.001** |
| 4 | 0.061 |
| 5 | **<0.001** |
| 6 | **0.012** |
| 7 | 0.565 |
